# Initial respiratory support modality and outcome in preterm infants with less than 32 weeks of gestation in China: A multicentre retrospective cohort study

**DOI:** 10.1111/ppe.12801

**Published:** 2021-08-24

**Authors:** Li Wang, Jia‐hui Li, Yong‐hui Yu, Lei Huang, Xiao‐yang Huang, Xiu‐fang Fan, Xiao‐hui Zhang, Chun‐lei Zhang, Qiang Liu, Ai‐rong Sun, Yong‐feng Zhang, Yang‐yang Cao, Ping Xu, Xiu‐xiang Liu, Jing‐cai Wu, Zhen‐ying Yang, Rong‐rong Sun, Xue‐yun Ren, Jing Li, Xiao‐li Wan, Bing‐ping Qiu, Shi‐ping Niu, Ren‐xia Zhu, Xiao‐kang Wang, Yi‐hui Zhang, Yan‐ling Gao, Li‐ping Deng, Jing Shi, Mei‐rong Bi

**Affiliations:** ^1^ The First Affiliated Hospital of Sun Yat‐sen University Guangzhou China; ^2^ Eyast Branch of Provincial Hospital Affiliated to Shandong University Jinan China; ^3^ The First Affiliated Hospital of Shandong First Medical University Jinan China; ^4^ Shandong Provincial Maternal and Child Health Care Hospital Cheeloo College of Medicine Shandong University Jinan China; ^5^ Qilu Hospital of Shandong University Jinan China; ^6^ Jinan Maternity and Child Healthcare Hospital Jinan China; ^7^ Yantai Yuhuangding Hospital Yantai China; ^8^ W.F. Maternal and Child Health Hospital Weifang China; ^9^ Linyi People's Hospital Linyi China; ^10^ Linyi Maternal and Child Health Care Hospital Linyi China; ^11^ Affiliated Hospital of Weifang Medical University Weifang China; ^12^ Taian Central Hospital Taian China; ^13^ Liaocheng People's Hospital Liaocheng China; ^14^ Binzhou Medical University Hospital Binzhou China; ^15^ Maternity and Child Health Care of Zaozhuang Zaozhuang China; ^16^ Taian Maternal and Child Health Care Hospital Taian China; ^17^ Dongying People's Hospital Dongying China; ^18^ Affiliated Hospital of Jining Medical College Jining China; ^19^ The Second Affiliated Hospital of Shandong First Medical University Jinan China; ^20^ Jinan second Maternity and Child Health Care Hospital Jinan China; ^21^ Tengzhou Central People's Hospital Tengzhou China; ^22^ Zibo Maternal and Child Health Hospital Zibo China; ^23^ Zibo Municipal Hospital Zibo China; ^24^ Central Branch of Provincial Hospital Affiliated to Shandong University Jinan China; ^25^ Juxian Peoples Hospital Rizhao China; ^26^ Dezhou People's Hospital Dezhou China; ^27^ Heze Municipal Hospital Heze China; ^28^ Liaocheng Second People's Hospital Liaocheng China; ^29^ Jinan Central Hospital Jinan China

**Keywords:** China, continuous positive airway pressure, neonatal intensive care unit, outcome, preterm infants

## Abstract

**Background:**

For initial respiratory management, continuous positive airway pressure (CPAP) is increasingly used for preterm infants, especially for gestational age less than 32 weeks. However, neonatologists are concerned about the potential risks of CPAP support failure.

**Objectives:**

To examine the association between different initial respiratory support modalities and the outcomes of preterm infants at <32 weeks of gestation across multiple neonatal intensive care units (NICU) in China.

**Methods:**

This study was carried out over a period of 12 months in 2018. Unadjusted relative risks (RR) for demographic and clinical characteristics were calculated for CPAP failure and CPAP success in the total cohort using log‐linear model based on generalised estimating equations for clustered observations.

**Results:**

Among 1560 preterm infants delivered at <32 weeks, the incidence of CPAP failure was 10.3%. After adjustment for demographic and clinical factors, the relative risk of mortality (RR 7.54, 95% CI 5.56, 10.44), pneumothorax (RR 9.85, 95% CI 2.89, 61.53), pulmonary haemorrhage (RR 7.78, 95% CI 4.51, 14.64) and BPD (RR 3.65, 95% CI 3.65, 4.51) were considerably higher for infants in the CPAP failure group than those in the CPAP‐S group. However, the risk of poor outcomes in CPAP failure infants was similar to that of those in the initial mechanical ventilation (MV) group.

**Conclusions:**

Continuous positive airway pressure failure was associated with an increased risk of mortality and major morbidities, including BPD, pulmonary haemorrhage and pneumothorax, and was comparable to the risk associated with initial MV.

**Synopsis**

**Study question**

The purpose of this study was to analyse data on initial respiratory management in preterm infants sourced from a large database in China.

**What's already known**

Data from several hospital‐based cohort studies strongly suggest that CPAP failure is associated with a higher risk of adverse outcomes.

**What this study adds**

This multicentre study of 28 NICUs in China shows that the incidence of CPAP failure was 10.3%. Strategies to promote successful CPAP application on infants with birthweight <1000 g, gestational age <30 weeks, maternal hypertension, surfactant use, caesarean and respiratory distress syndrome should be pursued energetically in China.

## 1 BACKGROUND

Respiratory distress syndrome (RDS) in newborns is the most common cause of morbidity and mortality and is an indication for ventilation in preterm infants.^1^ In recent years, the widespread implementation of nasal continuous positive airway pressure (CPAP) as the initial means of respiratory support for preterm infants has fundamentally changed respiratory management in the first hours of life. The universal use of CPAP has reduced the need for endotracheal intubation and mechanical ventilation (MV)^2^ and their associated lung injuries.^3^, ^4^ The American Academy of Pediatrics^5^ and the European Consensus Guidelines for the Management of RDS^6^ recommended that the initial application of CPAP be considered as the optimal mode of respiratory support.

A concern regarding the universal application of CPAP among preterm infants is that those with significant RDS may have a significant rate of CPAP failure, ultimately requiring intubation and starting on MV. Data from several hospital‐based cohort studies strongly suggest that CPAP failure is associated with a higher risk of adverse outcomes, including pneumothorax, bronchopulmonary dysplasia (BPD) and intraventricular haemorrhage (IVH), than that associated with the group for whom CPAP successfully reduces the need for intubation.^7^, ^8^, ^9^, ^10^, ^11^, ^12^

If universal CPAP is used, early identification of infants with subsequent CPAP failure is crucial. Research to date has reported numerous factors associated with CPAP failure, but it is not clear which predictors are most strongly correlated.^12^ For example, several observational studies^7^, ^8^, ^9^, ^10^, ^11^, ^12^ have reported that immature gestational age (gestational age), lower birth weight, male sex and fraction of inspired oxygen (FiO_2_) are associated with CPAP failure. A multicentre study^13^ reported that FiO_2_ in the second hour of life is a strong predictor of CPAP failure.

China is a middle‐income country with a large population density, while the low proportion of medical staff per capita is a real issue in the country. The number of physicians, number of nurses, nurse to bed ratio, physician to nurse ratio, proportion of physician with graduate degree and proportion of nurses with at minimum a college certificate is significantly inadequate, in China.^14^ Accordingly, there are some practical difficulties in the implementation of European or American guidelines for the management of RDS in China, and large sample and multicentre studies on the relationship between initial respiratory support pattern and prognosis are rare in China. We undertook this study to examine initial respiratory management patterns among preterm infants in China. The aims of the study were (i) to examine the incidence of CPAP failure and (ii) to compare neonatal outcomes within the CPAP failure group with those of infants in the CPAP success group and those in the initial MV group.

## 2 METHODS

This study is a retrospective cohort study. Uniformed neonatal cooperative research platforms were established in January 2018. Uniformed neonatal cooperative research platforms were established for all collaborating sites to ensure standardisation of clinical data and consistency of data collection methods. The initial CPAP support and CPAP failure, mortality incidence and morbidity data of preterm infants born in 28 level‐III NICUs in China were collected prospectively. The database provided maternal, delivery and neonatal data until the first NICU discharge, and the data were collected by trained staff using a standardised operating procedure.^15^

### 2.1 Cohort selection

The study population included all preterm infants with a gestational age <32 weeks who were admitted to the NICUs of 28 level‐III hospitals in China in 2018. Preterm infants who were out born, families who withdrew treatment due to socio‐economic factors and infants with missing initial respiratory management data were excluded. We also excluded infants with a congenital anomaly likely to affect respiratory function or early management and no requirement for respiratory support in the first 24 h.

The initial management with CPAP was defined as receiving a CPAP trial of at least a 30‐min duration. For all very low birth weight infants with risk factors for RDS, CPAP was given within 30 min after birth, for example infants with gestational age <30 weeks without endotracheal intubation were routinely given non‐invasive positive pressure ventilation after birth.^6^ Infants failing CPAP within the first 72 h of life were considered to experience CPAP failure and were intubated and started on MV.^9^, ^10^ Standards of mechanical ventilation for endotracheal intubation refer to Chinese guidelines for neonatal resuscitation (revised in 2016).^16^ Each centre chose the initial respiratory support mode according to the European RDS consensus guidelines.^6^ Following resuscitation and stabilisation at delivery, all infants (gestational age <32 weeks) were divided into three groups based on their initial respiratory support modality and its outcome at 72 h of age: the initial mechanical ventilation (MV) group, the CPAP failure (CPAP‐F) group and the CPAP success (CPAP‐S) group.

### 2.2 Data collection

The maternal variables included diabetes, hypertension, premature rupture of the membranes (PPROM) (>24 h) and caesarean. The neonatal variables included multiple birth (twin and above), sex, gestational age, birthweight, small for gestational age (birthweight below the 10th percentile for gestational age on a Fenton growth chart),^17^ surfactant use, and 1‐min and 5‐min Apgar scores <7. The incidence of the following poor outcomes was ascertained in the three groups: mortality, pneumothorax, pulmonary haemorrhage, severe/moderate BPD (need for supplemental oxygen or any form of positive pressure respiratory support at 36 weeks corrected gestational age),^18^ severe intraventricular haemorrhage (IVH; grades III and IV),^1^ necrotising enterocolitis (NEC; modified Bell stage II or greater),^1^ late‐onset neonatal sepsis (LOS)^19^, ^20^, ^21^ and retinopathy of prematurity (ROP; greater than stage 2).^1^

### 2.3 Statistical analysis

Demographic data are expressed as the mean (with standard deviation [SD]) or percentages. We performed inverse probability of treatment weighting (IPTW) to account for the potential influence of bias due to missing data in a logistic regression model framework.^22^ IPTW is adjusted with all observed objects as the ‘standard population’. To avoid the problem of extreme weights, we construct stabilised weight.^23^

Unadjusted relative risks (RR) for the demographic and clinical characteristics were calculated for CPAP failure and CPAP success in the total cohort using log‐linear model. Variables with unadjusted RR greater than 1.10 or less than 0.90 were retained in multivariable log‐linear model, and adjusted RR and 95% confidence interval (CI) were calculated. The simplified log‐liner model included all variables, and the interaction terms between CPAP and other variables were considered. To account for the heterogeneity in outcomes, we fit a random intercept regression model with NICU as the random intercept term.

R software version 4.0.2 was used to perform all the analysis (https://www.r‐project.org/). Package ‘lme4’ was used to fit log‐liner mixed‐effects models. Package ‘survey’ was used for IPTW.

### 2.4 Missing data

Less than 5% of the sample had missing covariate information requiring imputation. Fifty‐nine (2.8%) infants were missing initial mode and duration of respiratory support. Missing data were accounted for the original cohort (*n* = 2043) based on IPTW analysis.

### 2.5 Sensitivity analyses

To address bias due to unmeasured or uncontrolled confounding, we undertook a sensitivity analysis to address unmeasured confounding through the *E*‐value method.^24^

### 2.6 Ethics approval

The Institutional Review Board of Shandong Provincial Hospital Affiliated with Shandong University approved the project (ethical approval number: LCYJ: NO. 2019‐004). Informed consent was signed by the legal guardian of all participants.

## 3 RESULTS

**FIGURE 1:**
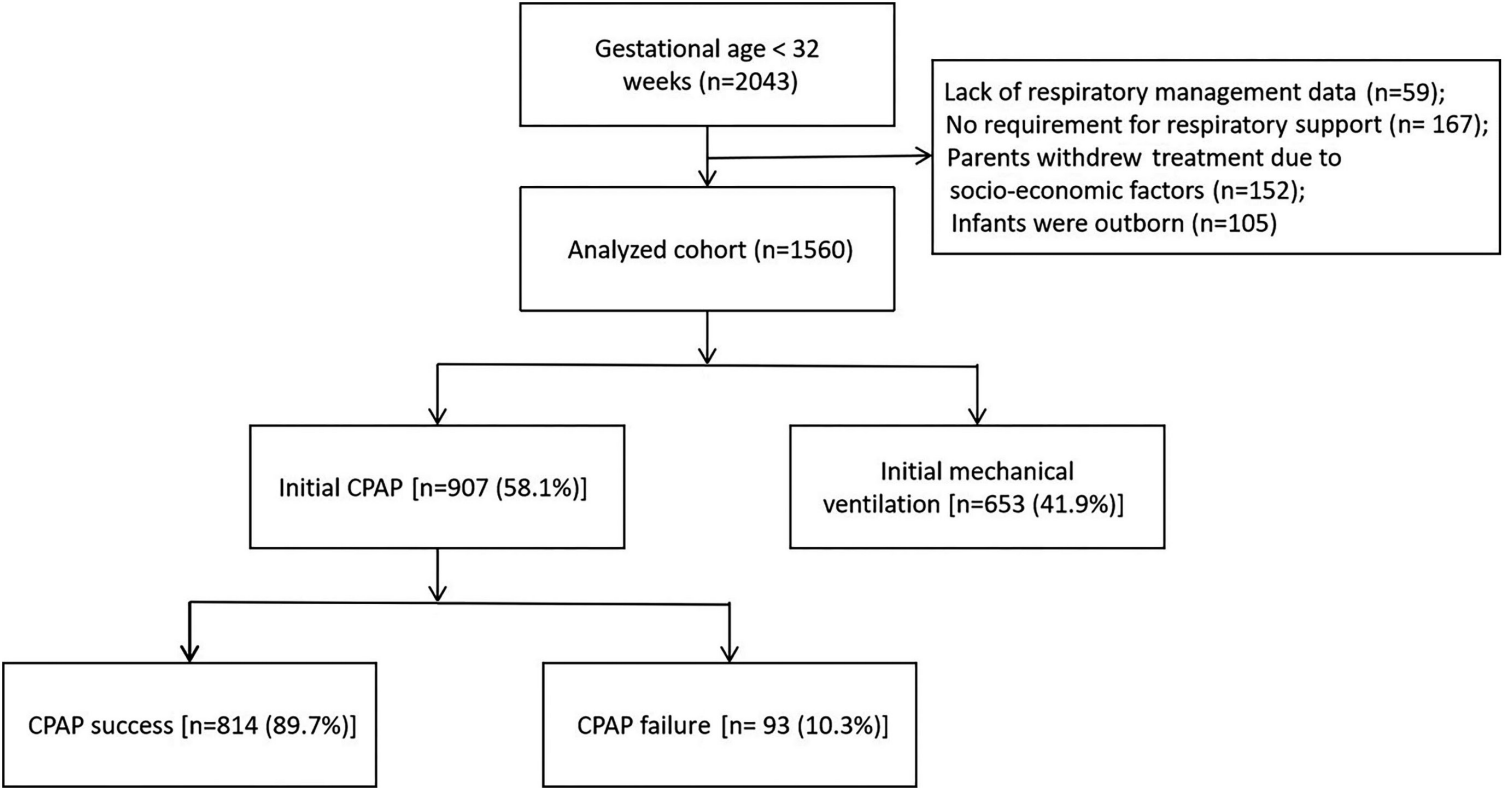
Flow diagram of the study population. A total of 2043 inborn infants born at a gestational age less than 32 weeks were enrolled in the study on their day of birth; 105 infants were excluded because they were out born. Additionally, 152 infants whose parents withdrew treatment due to socio‐economic factors, 167 infants with no requirement for respiratory support in the first 24 h and 59 infants who had missing initial respiratory management data were excluded

The 28 participating hospitals included 20 general hospitals and eight maternal and child healthcare hospitals, with an average of 59 and 40 beds in the neonatology departments and NICUs, respectively. A total of 2043 inborn infants born at a gestational age less than 32 weeks were enrolled in the study on their day of birth; 105 infants were excluded because they were out born. Additionally, 152 infants whose parents withdrew treatment due to socio‐economic factors, 167 infants with no requirement for respiratory support in the first 24 h and 59 infants who had missing initial respiratory management data were excluded. The remaining 1560 infants were included in this analysis, and of which, 653/1560 (41.9%) infants were in the initial MV group, while 907/1560 (58.1%) were in the CPAP group; the latter including 93/907 (10.3%) infants in the CPAP‐F group, 814/907 (89.7%) infants in the CPAP‐S group and (Figure 1). The final cohort had a mean (SD) birthweight of 1317 (318) g and gestational age of 29.3 (1.6) weeks.

### 3.1 Risk factors

**FIGURE 2:**
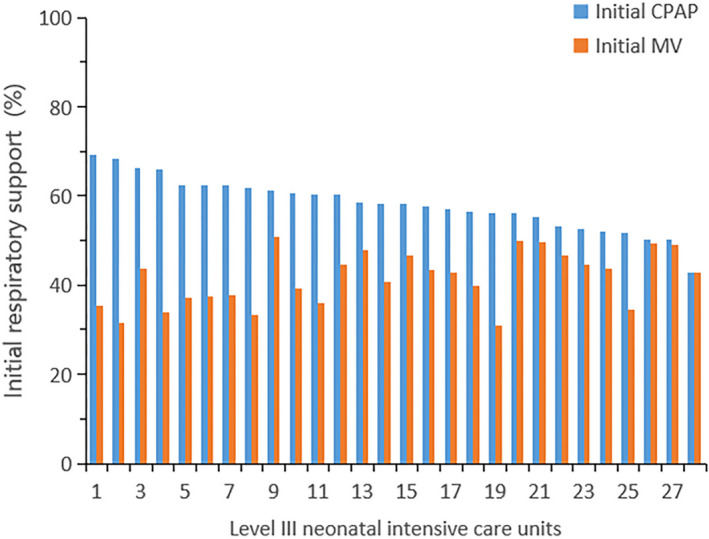
The proportion of initial respiratory support among centres

The proportions of initial respiratory support were fairly similar across centres (Figure 2). The success rate of CPAP ranged between 62.5% and 97.1% across the level‐III NICU's. Compared with infants for whom CPAP was successful, infants in the CPAP failure group had a higher incidence of birth weight <1000 g and gestational age <30 weeks, a lower incidence of surfactant use, a higher rate of maternal hypertension and delivery by caesarean without labour, a higher incidence of RDS and a somewhat lower Apgar score at 1 min (Table 1). It was found after adjusted that maternal hypertension, caesarean, gestational age <30 weeks, birthweight <1000 g, surfactant use, lower Apgar score at 1 min and RDS were associated with CPAP failure (Table 2).

**TABLE 1:** Univariate risk analysis of associations of demographic and clinical characteristics with CPAP failure

	CPAP success *n* = 814, *n* (%)	CPAP failure *n* = 93, *n* (%)	RR (95% CI)^a^
Maternal and neonatal variables			
Maternal hypertension	241 (29.6)	41 (44.1)	4.13 (2.65, 6.51)
Diabetes	108 (13.3)	18 (19.4)	1.57 (0.88, 2.67)
Premature rupture of membrane	184 (22.6)	17 (18.3)	0.76 (0.26, 1.29)
Caesarean	582 (71.5)	78 (83.9)	3.72 (1.94, 8.06)
Twins/triplets	158 (19.4)	17 (18.3)	0.93 (0.52, 1.58)
Male	433 (53.2)	53 (70.0)	1.17 (0.75, 1.81)
Small for gestational age	84 (10.3)	14 (15.1)	1.54 (0.81, 2.76)
Gestational age <30 weeks	295 (36.2)	45 (48.3)	1.36 (1.08, 1.71)
Birth weight <1000 g	58 (7.1)	14 (15.1)	2.11 (1.23, 3.64)
Surfactant use	294 (36.1)	20 (21.5)	1.29 (0.92, 1.81)
Apgar score at 1 min <7	161 (19.8)	32 (34.4)	4.92 (3.17, 7.70)
Apgar score at 5 min <7	32 (3.9)	8 (8.6)	2.30 (0.96, 4.92)
Respiratory distress syndrome	606 (74.4)	84 (90.3)	3.20 (1.67, 6.95)
Neonatal outcomes			
Mortality	43 (5.3)	19 (20.4)	4.60 (2.51, 8.21)
Pneumothorax	2 (0.2)	3 (3.2)	13.53 (2.21, 103.76)
Pulmonary haemorrhage	11 (1.4)	15 (16.1)	14.04 (6.27, 32.38)
Bronchopulmonary dysplasia	142 (17.4)	35 (37.6)	2.86 (1.79, 4.49)
Intraventricular haemorrhage	133 (16.3)	20 (21.5)	1.40 (0.81, 2.34)
Necrotising enterocolitis	18 (2.2)	3 (3.2)	1.47 (0.34, 4.46)
Late‐onset neonatal sepsis	201 (24.7)	25 (26.9)	0.84 (0.49, 1.38)
Retinopathy of prematurity	54 (6.6)	10 (10.8)	1.69 (0.79, 3.32)

Abbreviations: CI, confidence interval; RR, relative risk.

^a^ Inverse probability of treatment weighting.

**TABLE 2:** Adjusted relative risks of independent variables associated with CPAP failure

	Adjusted RR^a^ (95% CI)	Sensitivity analyses	
	CPAP failure	*E*‐value (RR)	*E*‐value (CI)
Maternal hypertension	4.13 (2.64, 6.46)	7.73	4.72
Caesarean	3.72 (1.84, 7.52)	6.90	3.08
Gestational age <30 wk	2.30 (1.34, 3.94)	4.03	2.01
Birth weight <1000 g	2.12 (1.56, 6.78)	3.66	2.49
Surfactant use	1.32 (1.45, 2.81)	1.97	2.26
Apgar score at 1 min <7	4.92 (3.16, 7.67)	9.31	5.77
Respiratory distress syndrome	3.20 (1.58, 6.48)	5.85	2.54

Note

Relative risks were compared with subjects with successful CPAP.

Abbreviations: BW, birth weight; CI, confidence interval; GA, gestational age; RDS, respiratory distress syndrome; RR, relative risk.

^a^ Adjusted relative risks with the following covariates: maternal hypertension, caesarean, surfactant use, gestational age, birth weight, Apgar score <7 at 1 min, respiratory distress syndrome.

### 3.2 Outcomes

The relative risk of mortality was 4.60 (95% CI 2.51, 8.21) for CPAP‐F group compared with CPAP‐S group. The risk of morbidities (i.e., pneumothorax, BPD and pulmonary haemorrhage) remained higher for CPAP‐F group compared with CPAP‐S group (Table 1). After adjustment for demographic and clinical factors, the relative risk of mortality, pneumothorax, pulmonary haemorrhage and BPD were considerably higher for infants in the CPAP‐F group than those in the CPAP‐S group (Table 3).

**TABLE 3:** Adjusted relative risks for the association of CPAP failure with adverse outcomes compared with infants who were those with CPAP success

	Adjusted RR^a^ (95% CI)	Sensitivity analyses	
	CPAP failure	*E*‐value (RR)	*E*‐value (CI)
Mortality	7.54 (5.56, 10.44)	14.56	10.59
Pneumothorax	9.85 (2.89, 61.53)	19.19	5.23
Pulmonary haemorrhage	7.78 (4.51, 14.64)	15.04	8.49
Bronchopulmonary dysplasia	3.65 (3.65, 4.51)	6.76	6.76
Intraventricular haemorrhage	2.74 (2.21, 3.42)	4.92	3.85
Necrotising enterocolitis	0.97 (0.52, 1.83)	1.21	1.00
Late‐onset neonatal sepsis	0.77 (0.62, 0.65)	1.92	2.45
Retinopathy of prematurity	0.63 (0.44, 0.93)	2.55	1.36

Note

Relative risks were compared with subjects with successful CPAP.

Abbreviations: CI, confidence interval; RR, relative risk.

^a^ Adjusted relative risks with the following covariates: maternal hypertension, caesarean, surfactant use, gestational age, birth weight, Apgar score <7 at 1 min, respiratory distress syndrome.

However, the risks of mortality and morbidity of BPD and pneumothorax for infants in the CPAP‐F group were similar to those for infants in the initial MV group (Table 4), in whom the incidence of BPD was 32.5% and the mortality rate was 26.5%.

**TABLE 4:** Adjusted relative risks for the association of Initial MV with adverse outcomes compared with infants who were those with CPAP failure

	Adjusted RR^a^ (95% CI)	Sensitivity analyses	
	Initial MV	*E*‐value (RR)	*E*‐value (CI)
Mortality	1.24 (0.99, 1.57)	1.79	1.00
Pneumothorax	0.56 (0.22, 1.32)	2.97	1.00
Pulmonary haemorrhage	2.01 (1.49, 2.73)	3.43	2.34
Bronchopulmonary dysplasia	1.01 (0.81, 1.26)	1.11	1.00

Note

Relative risks were compared with subjects with unsuccessful CPAP.

Abbreviations: CI, confidence interval; MV, mechanical ventilation; RR, relative risk.

^a^ Adjusted relative risks with the following covariates: maternal hypertension, caesarean, surfactant use, gestational age, birth weight, Apgar score <7 at 1 min, respiratory distress syndrome.

**FIGURE 3:**
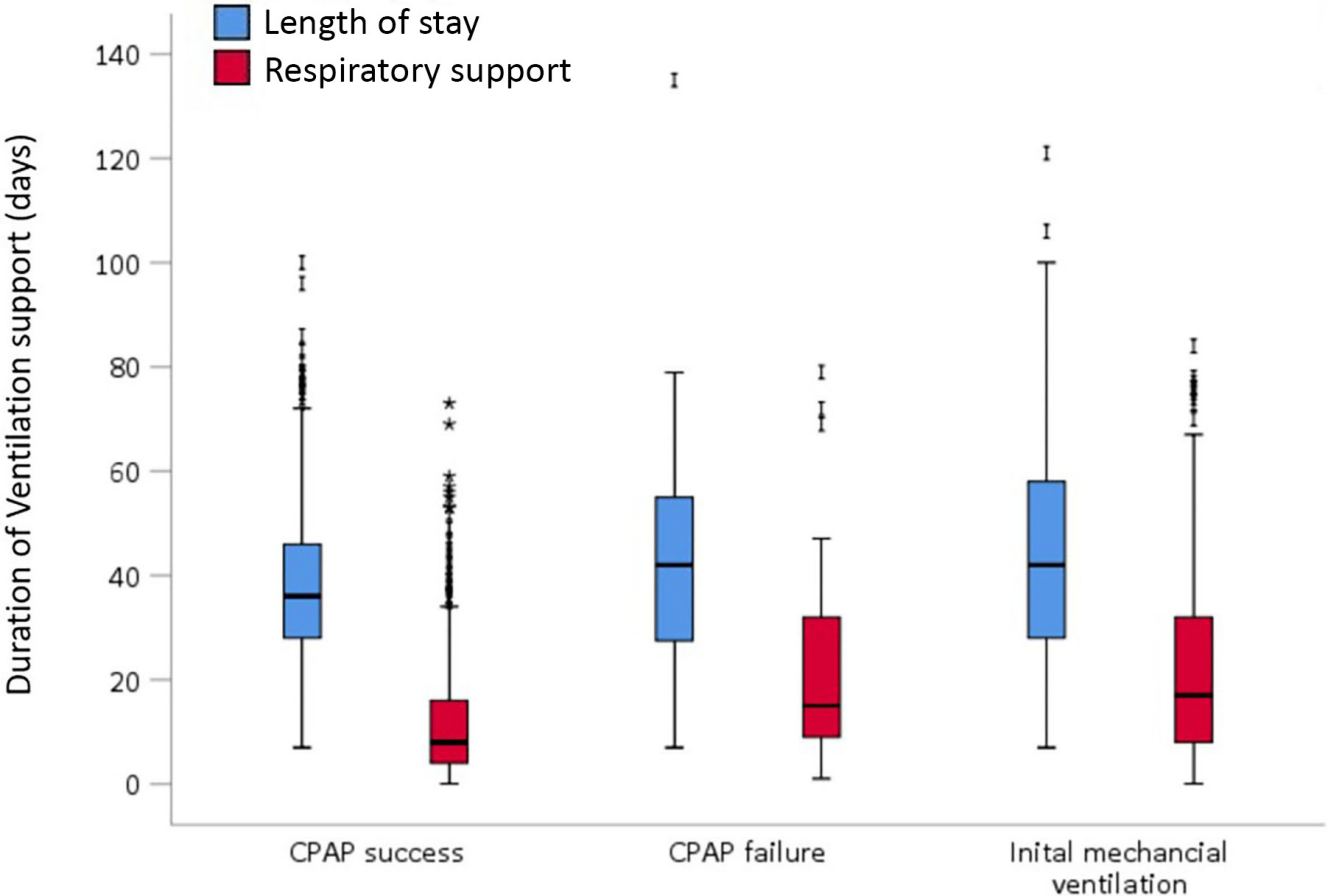
Outcomes: Respiratory support and length of stay, when infants who died, were excluded. Box and whisker plots of cumulative duration of respiratory support and length of stay. Whiskers denote 5th and 95th percentiles. All values differed between the CPAP‐S and CPAP‐F groups

When infants who died were excluded, the duration of respiratory support and length of hospital stay of infants in the CPAP‐F group were comparable to those of infants in the initial MV group (Figure 3). However, infants with unsuccessful CPAP had a prolonged duration of respiratory support (CPAP‐S median 8 [IQR 4–16] days vs. CPAP‐F 15 [9–32] days) and length of stay (CPAP‐S median 36 [IQR 28–46] days vs. CPAP‐F 42 [26.8–55] days) compared to the CPAP‐S group.

### 3.3 Sensitivity analyses

Results from sensitivity analyses were similar to those from the primary analysis. Sensitivity analysis showed that unknown confounders were not strongly associated with exposure factors and outcomes.

## 4 COMMENT

### 4.1 Principal findings

We found that the incidence of CPAP failure was 10.3% in this multicentre cohort study in China. In this study, results suggest that maternal hypertension, caesarean, gestational age <30 weeks, birthweight <1000 g, surfactant use, lower Apgar score at 1 min and RDS were associated with CPAP failure. CPAP failure was associated with an increased risk of mortality and major morbidities, including BPD, pulmonary haemorrhage and pneumothorax, and was comparable to the risk associated with initial MV. Infants with CPAP failure had a considerably prolonged duration of respiratory support and length of stay.

### 4.2 Strengths of the study

This study had several strengths. Our findings are based on a large multicentre cohort with limited missing data, and as such the results are representative of these units in China. We performed an inverse probability of treatment weighting to account for the potential bias due to missing data.^22^, ^23^

### 4.3 Limitations of the data

The study has a few limitations. First, some clinical information was not available. For example, not only did we not know the detailed information of the CPAP therapy of each centre, including the fraction of inspired oxygen (FiO_2_) and CPAP pressure change, but we were unable to evaluate the policies for respiratory support management in early life at each centre. Second, no data on radiologic severity of RDS were recorded. Finally, the study lacks information on CPAP device and type of nasal interface used of all the enrolled preterm infants.

### 4.4 Interpretation

Although CPAP is often effective for initial respiratory support,^2^, ^3^, ^4^, ^5^, ^6^ some preterm infants encounter CPAP failure and require endotracheal intubation and receive mechanical ventilation in the first 72 h after birth. We found that the incidence of CPAP failure was 10.3% in this multicentre cohort study in China. Rocha et al.^25^ reported that CPAP failure occurred in 20.6% (*n* = 131) patients. In 2013, Dargaville et al.^10^ reported that approximately 22% of newborns with gestational age <32 weeks were unsuccessful with CPAP. Subsequently, in 2016, Dargaville et al.^9^ reported that the proportion of infants with CPAP failure was 25% in a multicentre retrospective study. In a study by Ammari et al.,^12^ approximately 24% of newborns with birthweight <1250 g was unsuccessful with CPAP. However, the incidence of CPAP failure in our study was lower, which may be due to the higher utilisation rate of initial mechanical ventilation (see Figure 2). In China, many hospitals have an imbalance between physicians and nurses. We found that some units had a higher success rate of CPAP, which might be because these units have a more acceptable ratio of doctors to nurses.

This study shows that infants with CPAP failure had higher mortality and morbidity of BPD. We hypothesise that both the respiratory antecedents of CPAP failure and the interventions imposed on infants after it has occurred contribute to the risk of mortality and the development of serious complications. The sensitivity analysis showed a very small association between unknown confounders and outcomes. In a multicentre study, Hameed et al.^26^ reported that CPAP failure was associated with early neonatal death and a longer length of hospital stay. Using Australian and New Zealand Neonatal Network data, a study^9^ reported that among 19,103 preterm infants (gestational age <32 weeks), CPAP failure in preterm infants was associated with an increased risk of mortality and major morbidities, including BPD. In an observational study, the rates of mortality and common premature morbidities were higher in the CPAP failure group than in the CPAP success group.^12^ Furthermore, in our cohort study, when infants who died were excluded, the duration of respiratory support and length of hospital stay were approximately equal in the CPAP failure group compared with the mechanical ventilation group. Infants failing CPAP had a longer duration of respiratory support and length of hospital stay than those in the successful CPAP group.

In addition, our study also found that CPAP failure was associated with an increased risk of pulmonary haemorrhage. This difference may be attributed to the type of nasal interface used, use of surfactant and CPAP device.^8^ The need for ventilation with positive pressure and oxygen leads to excessive alveolar distension, causing stress damage to the alveolar capillaries, thereby contributing to the genesis of pulmonary haemorrhage.^27^ This result may be similar to the mechanism of pneumothorax reported previously.^10^ In addition, the number of physicians, number of nurses, nurse to bed ratio, physician to nurse ratio, proportion of physicians with post‐graduate degree and proportion of nurses with at minimum a college certificate is inadequate in China, and it is often difficult to provide better quality care.

While the results of our study may imply that mechanical ventilation should be initiated more readily in extremely preterm infants, caution is needed as our study design was purely observational and retrospective, with no control exercised over the selection of infants for inclusion or the intervention applied. Randomised controlled trials have demonstrated that the risk of adverse outcomes in those failing CPAP were similar to those in the initial MV group.^9^, ^10^ The initial MV group is undoubtedly different to those who received CPAP and includes infants requiring intubation in the delivery room for resuscitation, for whom initial CPAP would not have been an option and who were in worse condition at birth. Despite the best intentions to maximise non‐invasive support, many small infants will initially require MV, and approximately half of those with gestational age less than 28 weeks will fail their first attempt at extubation, with these infants having higher mortality and morbidity rates.^28^ The next step is to focus on implementing strategies to reduce CPAP failure.

We also found that characteristics overrepresented among infants failing CPAP included perinatal factors such as maternal hypertension, caesarean, gestational age <30 weeks, birthweight <1000 g, surfactant use, lower Apgar score at 1 min and RDS. Infants with these risk factors are more likely to have CPAP failure. Hameed et al.^26^ reported that CPAP failure was associated with birthweight ≤1500 g. Additionally, Dargaville et al.^10^ reported a strong association between caesarean and risk of CPAP failure and some evidence of increased risk in preterm infants with 25–28 weeks of gestation. In a study by Boo et al.,^29^ moderate RDS was one predictor of failure of CPAP. We also found that maternal hypertension was independently associated with CPAP failure. In China, for pregnant women with hypertension, obstetricians routinely use magnesium sulphate as a tocolytic agent. This could result in an increase in the CPAP failure rates due to the side effects that hypermagnesaemia can cause in the newborns, such as myorelaxation and respiratory depression.^30^

## 5 CONCLUSIONS

Continuous positive airway pressure failure was associated with an increased risk of mortality and major morbidities or poor outcomes, including BPD and pulmonary haemorrhage. Strategies to promote successful CPAP application on infants with birth weight <1000 g, gestational age <30 weeks, maternal hypertension, a lower incidence of surfactant use, caesarean, lower Apgar score at 1 min and RDS should be pursued energetically in China. Future studies should focus on comparison data before and after any recommended practice changes are implemented to evaluate effectiveness in improving neonatal CPAP success and outcomes. The collaboration group also needs to develop detailed CPAP use strategies, especially for infants with birthweight <1000 g, including timing and dosage of surfactant to promote the success of CPAP.

## Data Availability

The data that support the findings of this study are available from the corresponding authors upon reasonable request.
